# Histopathological tumour microenvironment score independently predicts outcome in primary operable colorectal cancer

**DOI:** 10.1002/2056-4538.12374

**Published:** 2024-04-22

**Authors:** Phimmada Hatthakarnkul, Kathryn Pennel, Peter Alexander, Hester van Wyk, Antonia Roseweir, Jitwadee Inthagard, Jennifer Hay, Ditte Andersen, Noori Maka, James Park, Campbell Roxburgh, Chanitra Thuwajit, Donald McMillan, Joanne Edwards

**Affiliations:** ^1^ School of Cancer Sciences University of Glasgow Glasgow UK; ^2^ Biomedical Science Program, Faculty of Medicine Siriraj Hospital Mahidol University Bangkok Thailand; ^3^ Academic Unit of Surgery University of Glasgow UK; ^4^ School of Medicine University of Glasgow Glasgow UK; ^5^ Glasgow Tissue Research Facility Queen Elizabeth University Hospital Glasgow UK; ^6^ Bioclavis Ltd Queen Elizabeth University Hospital Glasgow UK; ^7^ Department of Pathology Queen Elizabeth Hospital Glasgow UK; ^8^ Department of Surgery Queen Elizabeth University Hospital Glasgow UK

**Keywords:** colorectal cancer, histopathology, tumour microenvironment, immune infiltration, stromal invasion, Glasgow microenvironment score, Klintrup–Mäkinen grade, tumour stroma percentage, tumour budding, haematoxylin and eosin staining, prognostic biomarker

## Abstract

Colorectal cancer (CRC) is a heterogenous malignancy and research is focused on identifying novel ways to subtype patients. In this study, a novel classification system, tumour microenvironment score (TMS), was devised based on Klintrup–Mäkinen grade (KMG), tumour stroma percentage (TSP), and tumour budding. TMS was performed using a haematoxylin and eosin (H&E)‐stained section from retrospective CRC discovery and validation cohorts (*n* = 1,030, *n* = 787). TMS0 patients had high KMG, TMS1 were low for KMG, TSP, and budding, TMS2 were high for budding, or TSP and TMS3 were high for TSP and budding. Scores were assessed for association with survival and clinicopathological characteristics. Mutational landscaping and Templated Oligo‐Sequencing (TempO‐Seq) profiling were performed to establish differences in the underlying biology of TMS. TMS was independently prognostic in both cohorts (*p* < 0.001, *p* < 0.001), with TMS3 predictive of the shortest survival times. TMS3 was associated with adverse clinical features including sidedness, local and distant recurrence, higher T stage, higher N stage, and presence of margin involvement. Gene set enrichment analysis of TempO‐Seq data showed higher expression of genes associated with hallmarks of cancer pathways including epithelial to mesenchymal transition (*p* < 0.001), IL2 STAT5 signalling (*p* = 0.007), and angiogenesis (*p* = 0.017) in TMS3. Additionally, enrichment of immunosuppressive immune signatures was associated with TMS3 classification. In conclusion, TMS represents a novel and clinically relevant method for subtyping CRC patients from a single H&E‐stained tumour section.

## Introduction

Colorectal cancer (CRC) is a heterogenous malignancy which accounts for the third most common cause of cancer‐related death worldwide. Tumour node metastasis (TNM) staging is the current clinical method used for determining patient prognosis and treatment options. TNM staging is now considered suboptimal as it does not account for tumour heterogeneity and cannot be used to predict response to targeted therapies such as checkpoint inhibitors [1]. Novel subtyping methods including the consensus molecular subtypes (CMS) and cancer cell intrinsic subtypes (CRIS) were recently developed to address this; however, these subtypes rely on complex transcriptional and mutational profiling not yet feasible for translation to routine diagnostics [2, 3].

Simpler methods of subtyping patients based on tumour pathology from haematoxylin and eosin (H&E)‐stained sections present an exciting approach for rapid translation to clinical practice. For example, Klintrup–Mäkinen grade (KMG) is a histpathological measure of inflammatory cell density at the tumour invasive front, which is independently prognostic in CRC [4]. Patients with immunologically hot tumours observe the best clinical outcomes and those with a low density of immune infiltration have the poorest survival times [5, 6]. KMG is associated with tumoral expression of checkpoint proteins and further research is required to elucidate if it could be used as a predictive biomarker for response to checkpoint inhibitors [7]. Although KMG is highly prognostic, the KMG‐low group represents a diverse patient population and further histopathological scores may be required to properly segregate disease.

In addition to scores which rely on inflammation within the tumour microenvironment (TME), there is growing evidence for an important role of the stroma in dictating outcome. Tumour‐stroma percentage (TSP), which assesses the volume of intra‐tumour stromal invasion, is an important predictor of prognosis in CRC [8]. Patients with high stromal invasion have significantly worse clinical outcomes [9, 10, 11]. The Glasgow microenvironment score (GMS) was developed in 2015 to combine KMG and TSP to form three independently prognostic groups of CRC [12]. It has been reported to stratify survival outcome in CRC patients [13]. GMS0 patients (high KMG) have the best survival outcomes, GMS1 (low KMG/low TSP) have intermediate prognosis, and GMS2 (high TSP) observe the shortest survival times. Importantly, GMS has recently been shown to predict response to the existing therapeutics, with GMS0 predicative of a better response to CAPOX (capecitabine and oxaliplatin) versus FOLFOX (bolus and infused fluorouracil with oxaliplatin) chemotherapy in the TransSCOT clinical trial [13]. This work highlights the potential for simple histopathological scoring methods to be rapidly adopted in clinical practice for the prediction of response to chemotherapy regimens.

The phenotypic subtypes were developed in 2017, incorporating an immunohistochemically defined Ki67 proliferation index to divide GMS1 into two groups [14]. Phenotypic subtypes were shown to predict survival, recurrence, and response to therapy, but the subtype with poorest outcome was classified using the same protocol as GMS2 [15]. Within the GMS2 phenotype, there is a degree of patient–patient heterogeneity, and incorporation of another histopathologically defined marker may more thoroughly segregate patient outcome and tumour biology.

Tumour budding (TB) is a histopathological scoring method that can be used to predict patient prognosis and is thought to be a surrogate marker for epithelial to mesenchymal transition (EMT) or invasion [16]. The presence of a high number of tumour buds is linked to reduced cancer‐specific survival (CSS), venous invasion, and high TNM stage in CRC [17]. Here we propose that incorporation of TB with KMG and TSP histopathological measures provides a superior prognostic score, the tumour microenvironment score (TMS), for segregating CRC disease. We hypothesised that a combination of low KMG, high TSP, and high TB would be associated with profoundly reduced survival time. It was also predicted that the TMS histopathological subtyping method would have additional associated phenotypes in terms of mutational and transcriptional tumour profiles. Differential expression between each TMS group was assessed through mutational and transcriptional profiling to unravel potential mechanisms underpinning these prognostic phenotypes.

## Methods

### Patient cohorts

#### Discovery cohort

A retrospective cohort of stage I–III CRC patients (*n* = 1030) undergoing surgical resection with curative intent at Glasgow Royal Infirmary, Western Infirmary, or Stobhill Hospital between 1997 and 2007 was utilised as the discovery cohort in this study. Patients were excluded if they died within 30 days of surgery or received neoadjuvant therapy. Tumours were staged using the fifth edition of the AJCC/UICC TNM method and followed up for at least 5 years post‐surgery. This study was approved by the West of Scotland Research Ethics Committee (16/WS/0207) and data are held within the Glasgow and Clyde Safe Haven (GSH/18/ON007).

#### Validation cohort

To conform to REMARK guidelines, an independent retrospectively collected validation cohort was utilised to confirm findings from the discovery cohort. A retrospective cohort of stage I–III CRC patients (*n* = 787) who underwent surgery with curative intent at Glasgow Royal Infirmary between 1997 and 2013 was used as a validation cohort. Patients were excluded if they died within 30 days of surgery or received neoadjuvant therapy. Patients were staged using the 5th edition of the AJCC/UICC TNM method and followed up for at least 5 years post‐surgery. This study was approved by the West of Scotland Research Ethics Committee (MREC/01/0/3) and data are held within the Glasgow and Clyde Safe Haven (GSH21ON009).

#### Histopathological scoring

Scoring of both cohorts was performed for KMG, TSP (AR and JP), and TB (HW and PH) using a H&E full face tumour section. KM grading was performed as previously described [4]. In brief, the level of inflammatory infiltrate was assessed at the tumour invasive edge and graded as absent (0), patchy (1), thin and continuous band (2), or thick continuous band (3) by a single observer in cohort 1 (JP) and cohort 2 (PA). Similarly, TSP was scored as previously described [8]. In brief, a representative intra‐tumour area was assessed for the percentage of stromal invasion at ×20 objective magnification with tumour cells present in all four corners of the field of view. Tumours with ≥50% stroma within the tumour were classified as high and those with <50% were classified as low for TSP. TB assessment was performed in the H&E‐stained full CRC section. Budding score was performed manually, and the highest budding counts per 0.785 mm^2^ were used to define budding status using an ×20 objective lens. Cases were classified as low (0–9 buds) or high (10+ buds) in accordance with international agreement [18].

#### Tumour microenvironment score

**Figure 1:**
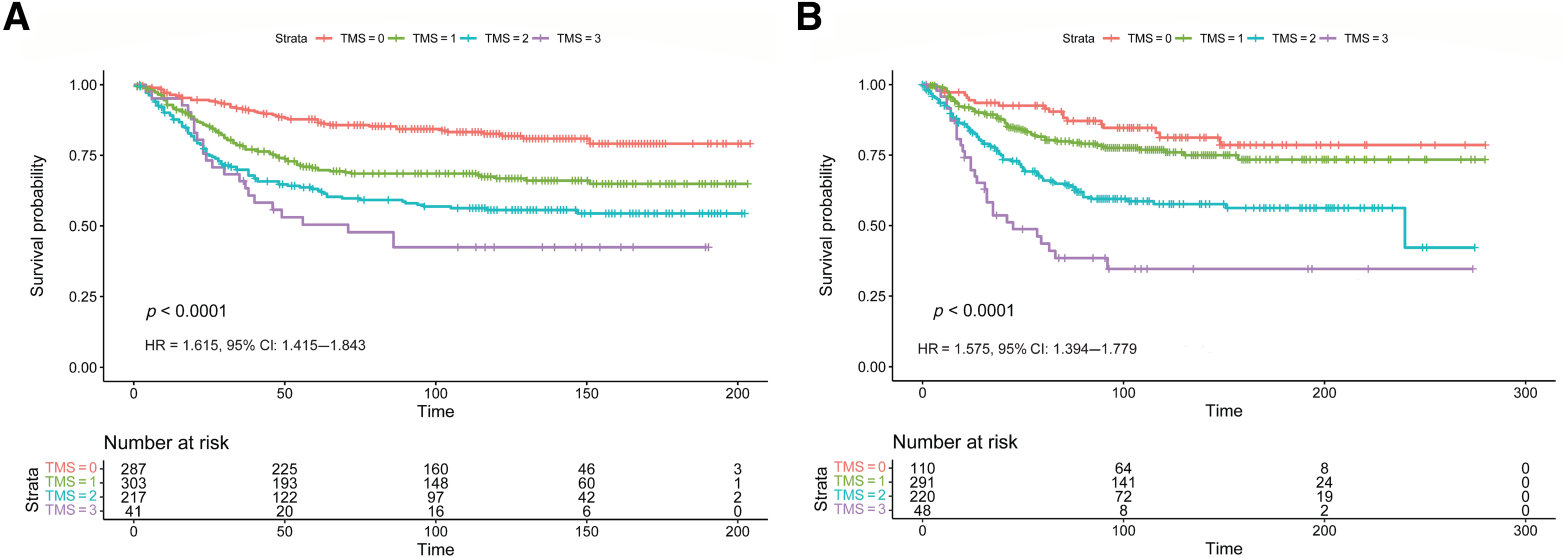
The association between TMS and CSS across two retrospective CRC cohorts. KM survival analysis showing the association between TMS and CSS in (A) the discovery cohort and (B) the validation cohort. Created in Adobe Acrobat 2020.

Measures of tumour histopathology can be used to predict patient outcomes and segregate disease phenotypes. In this article, we have identified a novel classification system, TMS, which histologically groups patients based on KMG, TSP, and TB. Representative images of the components of the TMS in H&E‐stained tumour resections are shown in Figure 1A. This method can be performed on a single H&E‐stained tumour resection to form four histologically distinct groups. Patients classified as high for KMG were assigned to TMS0, patients low for KMG, TSP, and TB were classified as TMS1, patients low for KMG, high for TSP, or high for TB were classified as TMS2, and patients low for KMG but high for both TSP and TB were classified as TMS3. The combination of these histopathological scores to form TMS is shown in supplementary material, Figure S1.

The overlap between TMS and previously validated subtypes (CMS and GMS) is shown in an alluvia plot in supplementary material, Figure S2 using data from cohort 2.

### Assessment of associated biological profiles of TMS groups

#### Mutational analysis

Panel mutational sequencing was performed on a subset of the discovery cohort (*n* = 157). DNA was extracted by Molecular Diagnostics NHS Tayside and quality was confirmed by Qubit assay (Thermo Fisher Scientific, Waltham, MA, USA). Sequencing was performed by Glasgow Precision Oncology Laboratory using a custom panel designed in house of 196 genes and a HiSeq4000 (Illumina, San Diego, CA, USA). The genes included in the panel are listed in supplementary material, Table S1.

#### Transcriptomic analysis

Single tissue sections from the discovery cohort (*n* = 59) of patients who had undergone resection for CRC were used for Templated Oligo‐Sequencing (TempO‐Seq) analysis using a whole transcriptome panel. In brief, formalin‐fixed paraffin‐embedded (FFPE) tissues were digested and deparaffinised. The lysate was then combined with detector oligonucleotides, which were annealed in immediate juxtaposition to each other on the targeted RNA template and ligated [19]. Amplification of ligated oligonucleotides was performed using a unique primer set for each sample, introducing a sample‐specific barcode and Illumina adaptors. Barcoded samples were pooled into a single library and run on an Illumina HiSeq 2500 High Output v4 flowcell and the sequencing reads were demultiplexed using BCL2FASTQ software (Illumina). FASTQ files were aligned to the human whole transcriptome v2.0 panel, which consists of 22,537 probes, using STAR [20]. Up to two mismatches were allowed in the 50‐nucleotide sequencing read. These data can be accessed at ArrayExpress at E‐MTAB‐13077.

#### Gene set enrichment analysis (GSEA)

TempO‐Seq counts were analysed using R package DESeq2. Two cohorts, the discovery cohort (*n* = 59) and the validation cohort (*n* = 29), were utilised. The normalised counts were then analysed through the GSEA program [21] (https://www.gsea-msigdb.org/gsea/msigdb/index.jsp). The molecular signature database (MSigDB) was employed to compare TMS subtypes using hallmark gene sets and immunologic signature gene sets. The enrichment pathways were determined based on the nominal *p* value and normalised enrichment score.

### Statistical analysis

Kaplan–Meier (KM) survival curves were utilised to determine association between TMS and CSS in SPSS v22 (IBM, Armonk, NY, USA). Univariate and multivariate Cox regression was performed to determine if TMS was independently prognostic. Chi‐squared tables of association were used to determine association between TMS and clinicopathological features. Non‐parametric Kruskal–Wallis tests were utilised to determine association between immune infiltrates and TMS with box plots constructed using GraphPad Prism version 9 (GraphPad Software, La Jolla, CA, USA). Mutational analyses were performed in R version 1.4 studio using the Maftools version 2.18.0 package. Gene expression counts from TempO‐Seq transcriptional profiling were normalised in R Studio using DESeq2 version 1.43.1 and analysed using GSEA application version 4.2.3. Significance was set to *p < 0.05 for KM, chi‐squared, non‐parametric Kruskal–Wallis tests, and GSEA.

## Results

### The TMS is prognostic in discovery and validation retrospective CRC patient cohorts

To determine if TMS represents a novel prognostic marker for CRC, two independent retrospective patient cohorts were utilised. After exclusion criteria were applied, there were 855 patients from the discovery cohort included in downstream analysis, as shown in the consort diagram (supplementary material, Figure S3). Of these cases, 291 were classified as TMS0, 306 as TMS1, 217 as TMS2, and 41 as TMS3. In the validation cohort, following exclusion criteria there were 671 patients included in the downstream analysis (supplementary material, Figure S4). Of these patients, 110 were classified as TMS0, 292 as TMS1, 221 as TMS2, and 48 as TMS3.

We performed KM survival analyses to test if TMS was associated with CSS in both cohorts. In the discovery cohort, TMS was significantly associated with CSS [hazard ratio (HR) = 1.615, 95% CI: 1.415–1.843, log rank *p* < 0.001] (Figure 1A). As hypothesised, patients classified as immune phenotype (TMS0) (*n* = 291) had a 5‐year survival of 86% compared to 69% in the TMS1 subtype (*n* = 306), 60% in TMS2 (*n* = 217), and only 48% in the invasive (TMS3) group (*n* = 41). The median survival time was 173 months for TMS0 patients, 145 months for TMS1 patients, 125 months for TMS2 patients dropping to 100 months for TMS3 patients. In the same cohort, the 5‐year survival of GMS0 patients was 77% dropping to 59% for GMS1 and 41% for GMS2 patients.

Similarly, in the validation cohort, KM survival analysis showed that TMS was significantly associated with CSS (HR = 1.575, 95% CI: 1.394–1.779, log rank *p* < 0.001) (Figure 1B). Patients classified as immune phenotype (TMS0) (*n* = 110) had a 5‐year survival of 72% compared to 71% in the TMS1 subtype (*n* = 292), 51% in TMS2 (*n* = 221), and only 31% in the invasive (TMS3) group (*n* = 48). The median survival was 233 months for TMS0 patients, 219 months for TMS1 patients, 168 months for TMS2 patients dropping to 116 months for TMS3 patients. The TMS builds upon GMS as the 5‐year survival of the worst prognostic group, GMS2, was 47% in this cohort, 67% for GMS1, and 74% for GMS0.

When individual variables were entered into univariable Cox regression from the discovery cohort, T stage, N stage, margin involvement, peritoneal involvement, venous invasion, modified Glasgow prognostic score (mGPS), GMS, and TMS were significantly prognostic as shown in Table 1. When these variables were taken forward into multivariable Cox regression, TMS remained independently associated with the CSS of patients with CRC (HR = 1.468, 95% CI: 1.256–1.715, log rank *p* < 0.001) (Table 1). These results indicate that TMS is an important prognostic factor that builds upon previous histopathological scoring systems and predicts the outcome irrespective of other commonly recorded clinicopathological measures. Concordantly, in the validation cohort, upon univariate Cox regression T stage, N stage, margin involvement, peritoneal involvement, venous invasion, mGPS, GMS, CMS, and TMS were significantly prognostic (Table 2). When these variables were assessed using multivariable Cox regression, TMS was independently prognostic (HR = 2.035, 95% CI: 1.517–2.730, log rank *p* < 0.001) as shown in Table 2.

**Table 1:** Cox regression showing the prognostic nature of clinicopathological features at univariate and multivariate levels for the discovery cohort

	Univariate analysis	*p*	Multivariate analysis	*p*
	HR		HR	
Age (<65/≥65 years)	1.193 (0.882–1.615)	0.247	–	–
Sex (male/female)	1.163 (0.878–1.540)	0.291	–	–
Tumour site (colon/rectum)	1.128 (0.834–1.525)	0.437	–	–
T stage (1/2/3/4)	1.848 (1.498–2.281)	**<0.001**	1.015 (0.690–1.577)	0.611
N stage (0/1/2)	2.168 (1.813–2.592)	**<0.001**	1.764 (1.352–2.218)	**<0.001**
MMR status (pMMR/dMMR)	0.732 (0.484–1.107)	0.124	–	–
Margin involvement (absent/present)	3.733 (2.393–5.821)	**<0.001**	1.457 (0.717–2.960)	0.155
Peritoneal involvement (absent/present)	2.602 (1.956–3.462)	**<0.001**	1.690 (1.166–2.448)	**<0.001**
Vascular invasion (absent/present)	2.211 (1.670–2.928)	**<0.001**	1.367 (0.946–1.974)	**0.003**
mGPS (0/1/2)	1.659 (1.360–2.025)	**<0.001**	1.660 (1.318–2.091)	**<0.001**
GMS (0/1/2)	2.059 (1.673–2.535)	**<0.001**	1.158 (0.766–1.750)	**<0.001**
TMS (0/1/2/3)	1.701 (1.429–2.025)	**<0.001**	1.468 (1.256–1.715)	**<0.001**

Bold font highlights significant *p* values of <0.05.

**Table 2:** Cox regression showing the prognostic nature of clinicopathological features at univariate and multivariate levels for the validation cohort

	Univariate analysis	*p*	Multivariate analysis	*p*
	HR		HR	
Age (<65/≥65 years)	1.292 (0.940–1.776)	0.114	–	–
Sex (male/female)	1.315 (0.966–1.791)	0.082	–	–
Tumour site (colon/rectum)	1.129 (0.810–1.574)	0.472	–	–
T stage (1/2/3/4)	1.882 (1.490–2.377)	**<0.001**	1.276 (0.999–1.629)	0.051
N stage (0/1/2)	2.089 (1.721–2.535)	**<0.001**	1.610 (1.334–1.942)	**<0.001**
MMR status (pMMR/dMMR)	0.715 (0.495–1.034)	0.075	–	–
Margin involvement (absent/present)	4.046 (2.581–6.342)	**<0.001**	2.584 (1.801–5.114)	**<0.001**
Peritoneal involvement (absent/present)	2.071 (1.510–2.841)	**<0.001**	1.423 (0.973–2.081)	0.069
Venous invasion (absent/present)	1.506 (1.110–2.043)	**0.008**	1.234 (0.847–1.799)	0.274
mGPS (0/1/2)	1.424 (1.181–1.717)	**<0.001**	1.159 (0.920–1.460)	0.211
GMS (0/1/2)	1.755 (1.356–2.271)	**<0.001**	0.714 (0.413–1.235)	0.229
TMS (0/1/2/3)	1.506 (1.314–1.725)	**<0.001**	1.379 (1.094–1.739)	**0.007**

Bold font highlights significant *p* values of <0.05.

Taken together, these data highlight the TMS as a novel independently prognostic marker for CRC, which could be easily translated into routine diagnostic pathology. The results build upon the former histopathological subtyping method GMS, as the Cox regression statistical testing proves TMS to be independently prognostic from GMS.

### The relationship between TMS and survival outcome is enhanced in rectal cancer

**Figure 2:**
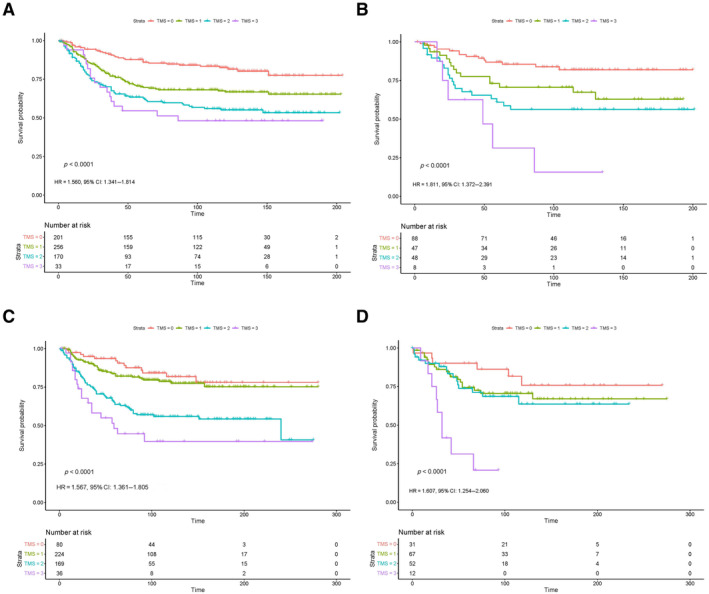
The association between TMS and CSS in colonic and rectal cases across two retrospective CRC cohorts. KM survival curves showing the association between TMS and CSS in the discovery cohort in (A) colon cancer cases and (B) rectal cancer cases. KM survival curves showing the association between TMS and CSS in the validation cohort in (C) colon cancer cases and (D) rectal cancer cases.

Next, we investigated the prognostic significance of TMS relative to tumour subsite. In the discovery cohort, KM survival analysis showed that TMS is associated with CSS in both colon (HR = 1.560, 95% CI: 1.341–1.814, *p* < 0.0001) and rectal cases (HR = 1.811, 95% CI: 1.372–2.391, *p* < 0.0001) (Figure 2A,B). However, the association of TMS3 with poorest survival time was enhanced in rectal cases with only 10% alive at 5 years post‐surgery (HR = 1.811, 95% CI: 1.372–2.391, log rank *p* < 0.001) (Figure 2B). In colon cases from the discovery cohort, the median survival time of TMS0 patients (*n* = 201) was 172 months, compared to 145 months for TMS1 (*n* = 256), 124 months for TMS2 (*n* = 170), and 108 months in TMS3 patients (*n* = 33). In rectal cases, the median survival time for TMS0 patients was 173 months (*n* = 88) compared to 139 months in TMS1 (*n* = 47), 128 months in TMS2 (*n* = 48), and 58 months in TMS3 cases (*n* = 8).

These trends were validated in the second cohort, and the association between TMS3 and reduced CSS in rectal cases was even more pronounced, with 16% of TMS3 patients alive 5 years post‐surgery (HR = 1.607, 95% CI: 1.254–2.060, *p* < 0.0001) (Figure 2C,D). Although not as pronounced, there was still a significant association between TMS and outcome in colon cases (HR = 1.567, 95% CI: 1.361–1.805, *p* < 0.0001). In colon cancer cases from the validation cohort, the median survival time of TMS0 patients (*n* = 80) was 235 months compared to 224 months for TMS1 (*n* = 224), 163 months for TMS2 (*n* = 169), and 129 months for TMS3 patients (*n* = 36). In rectal cancer cases, the median survival time for TMS0 patients was 220 months (*n* = 31) compared to 199 months in TMS1 (*n* = 67), 165 months in TMS2 (*n* = 52), and 44 months in TMS3 cases (*n* = 12).

These data again prove the superiority of TMS over GMS due to the significant association with prognosis not only in colon cancer but also in rectal cancer.

### TMS is associated with clinicopathological features and distinct patterns of immune cell infiltration

When TMS was assessed for relationship with clinicopathological characteristics in the discovery cohort, chi‐squared analysis showed a significant association with tumour subsite (*p* = 0.003), recurrence (*p* < 0.001), T stage (*p* < 0.001), N stage (*p* < 0.001), margin involvement (*p* = 0.006), peritoneal involvement (*p* < 0.001), and vascular invasion (*p* = 0.004) (Table 3). Patients with TMS3 disease were more likely to have left‐sided disease, local and distant recurrence, higher T stage, higher N stage, and more margin involvement (supplementary material, Figure S5A). In the validation cohort, similar results were found that TMS was associated with recurrence (*p* < 0.001), T stage (*p* < 0.001), N stage (*p* < 0.001), marginal involvement (*p* < 0.001), vascular invasion (*p* = 0.001), and peritoneal involvement (*p* < 0.001) (Table 4). In concordance with the discovery cohort, TMS3 was indicative of higher rates of recurrence, higher T stage, higher N stage, more margin involvement, venous invasion, and peritoneal involvement (supplementary material, Figure S5B). Interestingly, there was no association between TMS and mismatch repair (MMR) status in either cohort and the distribution across TMS of MMR proficient and MMR deficient cases is shown in supplementary material, Figure S6.

**Table 3:** The association between TMS and clinical characteristics based on chi‐squared analysis in the discovery cohort

Clinical characteristic	TMS				*p*
	0	1	2	3	
Age					
≥65	88 (30.2)	83 (27.1)	81 (37.3)	17 (41.5)	**0.041**
<65	203 (69.8)	223 (72.9)	136 (62.7)	24 (58.5)	
Sex					
Male	148 (50.9)	159 (52.0)	102 (47.0)	17 (41.5)	0.472
Female	143 (49.1)	147 (48)	115 (53.0)	24 (58.5)	
Tumour subsite					
Right colon	118 (40.8)	136 (44.7)	97 (45.3)	20 (48.8)	**0.003**
Left colon	89 (30.8)	124 (40.8)	71 (33.2)	15 (36.6)	
Rectum	82 (28.4)	44 (14.5)	46 (21.5)	6 (14.6)	
Recurrence					
None	176 (86.3)	186 (72.9)	103 (62.8)	11 (40.7)	**<0.001**
Local	4 (2)	10 (3.9)	10 (6.1)	2 (7.4)	
Distant	24 (11.8)	59 (23.1)	51 (31.1)	14 (51.9)	
T stage					
1	27 (9.3)	5 (1.6)	7 (3.2)	0 (0)	**<0.001**
2	62 (21.3)	25 (8.2)	19 (8.8)	0 (0)	
3	144 (49.5)	188 (61.4)	108 (49.8)	25 (61.0)	
4	58 (19.9)	88 (28.8)	83 (38.2)	16 (39.0)	
N stage					
0	208 (71.7)	197 (64.6)	115 (53.5)	16 (39.0)	**<0.001**
1	67 (23.1)	69 (22.6)	67 (31.2)	16 (39.0)	
2	15 (5.2)	39 (12.8)	33 (15.3)	9 (22.0)	
MMR status					
pMMR	224 (77.5)	251 (84.8)	176 (83.0)	35 (85.4)	0.115
dMMR	65 (22.5)	45 (15.2)	36 (17.0)	6 (14.6)	
mGPS					
0	144 (57.6)	109 (47.2)	95 (54.0)	16 (55.2)	0.411
1	67 (26.8)	73 (31.6)	48 (27.3)	9 (31.0)	
2	39 (15.6)	49 (21.2)	33 (18.8)	4 (13.8)	
Margin involvement					
Absent	286 (98.3)	290 (94.8)	200 (92.2)	37 (90.2)	**0.006**
Present	5 (1.7)	16 (5.2)	17 (7.8)	4 (9.8)	
Vascular invasion					
Absent	218 (74.9)	205 (67.0)	130 (59.9)	26 (63.4)	**0.004**
Present	73 (25.1)	101 (33.0)	87 (40.1)	15 (36.6)	
Peritoneal involvement					
Absent	39 (82.1)	220 (71.9)	134 (61.8)	25 (61.0)	**<0.001**
Present	52 (17.9)	86 (28.1)	83 (38.2)	16 (39.0)	

Bold font highlights significant *p* values of <0.05.

**Table 4:** The association between TMS and clinical characteristics based on chi‐squared analysis in the validation array

Clinical characteristic	TMS				*p*
	0	1	2	3	
Age					
<65	42 (37.8)	96 (32.9)	63 (28.5)	16 (33.3)	0.566
65–74	30 (27)	91 (31.2)	81 (36.7)	17 (35.4)	
≥75	39 (35.1)	105 (36)	77 (34.8)	15 (31.3)	
Sex					
Male	49 (44.5)	140 (47.9)	92 (41.7)	20 (41.7)	0.517
Female	62 (55.9)	152 (52.1)	129 (58.3)	28 (58.3)	
Tumour subsite					
Right colon	38 (34.2)	127 (43.5)	100 (45.2)	19 (39.6)	0.655
Left colon	42 (37.8)	97 (33.2)	69 (31.2)	17 (35.4)	
Rectum	31 (27.9)	68 (23.3)	52 (23.5)	12 (25)	
Recurrence					
None	87 (83.7)	206 (75.7)	131 (62.1)	19 (44.2)	**<0.001**
Local	5 (4.8)	10 (3.7)	16 (7.6)	8 (18.6)	
Distant	12 (11.4)	47 (17.3)	54 (25.6)	13 (30.2)	
Both local/distant	1 (1.0)	9 (3.3)	10 (4.7)	3 (7)	
T stage					
1	7 (6.3)	16 (5.5)	4 (1.8)	0 (0)	**<0.001**
2	28 (25.2)	26 (8.9)	15 (6.8)	1 (2.1)	
3	61 (55.0)	181 (62)	114 (51.6)	17 (35.4)	
4	15 (13.5)	69 (23.6)	88 (39.8)	30 (62.5)	
N stage					
0	87 (78.4)	185 (63.4)	110 (49.8)	20 (41.7)	**<0.001**
1	20 (18.0)	80 (27.4)	79 (35.7)	18 (37.5)	
2	4 (3.6)	27 (9.2)	32 (14.5)	10 (20.8)	
MMR status					
pMMR	76 (69.7)	193 (68.2)	139 (64.1)	28 (62.2)	0.644
dMMR	17 (15.6)	43 (15.2)	46 (21.2)	10 (22.2)	
mGPS					
0	68 (61.3)	181 (62.0)	127 (57.5)	24 (50.0)	0.609
1	22 (19.8)	68 (23.3)	55 (24.9)	13 (27.1)	
2	21 (18.9)	43 (14.7)	39 (17.6)	11 (22.9)	
Margin involvement					
Absent	110 (99.1)	279 (95.5)	203 (91.9)	39 (81.3)	**<0.001**
Present	1 (0.9)	13 (4.5)	18 (8.1)	9 (18.8)	
Venous invasion					
Absent	64 (57.7)	151 (51.7)	88 (39.8)	15 (31.3)	**0.00**1
Present	47 (42.3)	141 (48.3)	133 (60.2)	33 (68.8)	
Peritoneal involvement					
Absent	98 (88.3)	230 (78.8)	144 (65.2)	20 (41.7)	**<0.001**
Present	13 (11.7)	62 (21.2)	77 (34.8)	28 (58.3)	

Bold font highlights significant *p* values of <0.05.

**Figure 3:**
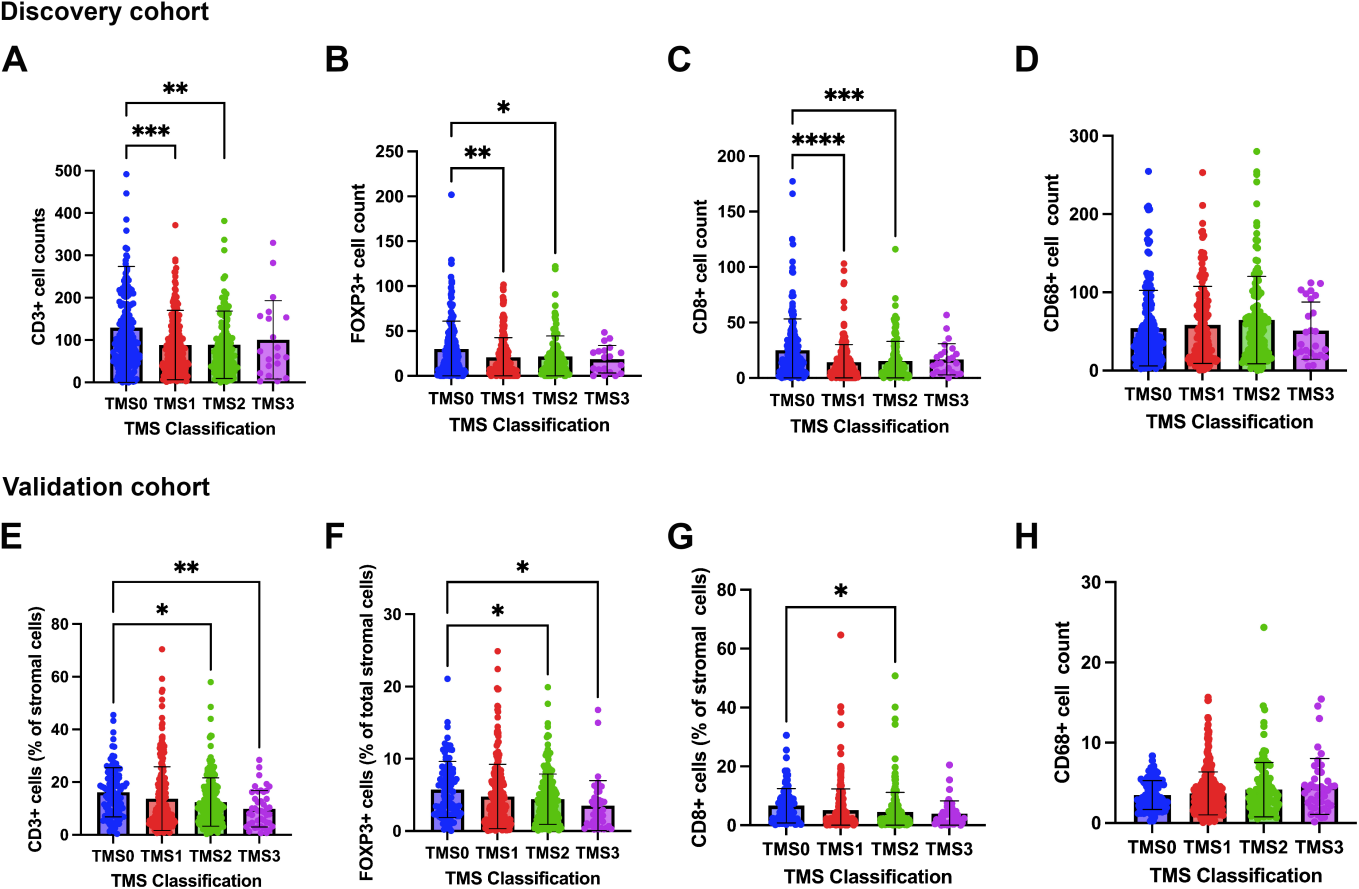
Immune profiles of TMS. Box plots showing (A) CD3+, (B) CD8+, (C) FOXP3+, and (D) CD68+ cell counts relative to TMS in the discovery cohort. Box plots showing (A) CD3+, (B) CD8+, (C) FOXP3+, and (D) CD68+ cell counts relative to TMS in the validation cohort. *p < 0.05; **p < 0.005; ***p < 0.001.

In the discovery cohort, when TMS was assessed for association with immune infiltrates, Kruskal–Wallis non‐parametric tests showed a significant association between CD3+ infiltrating lymphocytes with reduced infiltration in TMS3 tumours (*p* = 0.005) (Figure 3A). A similar trend was observed for CD8+ cytotoxic T cells (*p* < 0.001) and FOXP3+ regulatory T cells (*p* = 0.001) (Figure 3B,C). There was no association between CD68+ macrophage infiltration and TMS (*p* = 0.198) (Figure 3D). In the validation cohort, CD3+ infiltration was significantly associated with TMS, with TMS3 tumours observing the lowest density of T cell infiltration (Figure 3E). CD8+ and FOXP3+ cell counts were also significantly associated with TMS (*p* < 0.001, *p* = 0.029, respectively) (Figure 3F,G). In concordance with data from the discovery cohort, the level of infiltrating macrophages (CD68+) was not associated with TMS subtype (*p* = 0.163) (Figure 3H). Additionally, when entered into multivariate Cox regression, TMS and immune infiltration were shown to be independently prognostic in both discovery and validation cohorts. These data show that TMS is predictive of patient outcome irrespective of infiltration of specific immune populations (supplementary material, Tables S2 and S3).

### Mutational and transcriptional profiling reveals differences in the underlying biology associated with TMS subtypes

**Figure 4:**
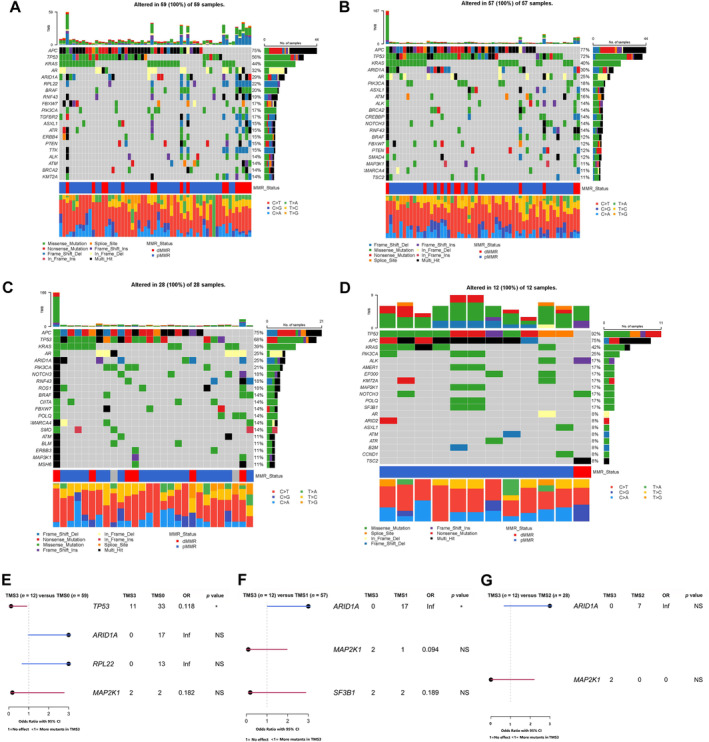
Mutational landscape of TMS. (A) Oncoplot showing the top mutated genes in TMS0 cases. (B) Oncoplot showing the top mutated genes among TMS1 cases. (C) Oncoplot showing the top mutated genes within TMS2 cases. (D) Oncoplot showing the top mutated genes within TMS3 cases. (E) Forest plot showing significantly differentially mutated genes across TMS0 and TMS3 cases. (F) Forest plot showing significantly differentially mutated genes across TMS1 and TMS3 cases. (G) Forest plot showing significantly differentially mutated genes across TMS2 and TMS3 cases. *p < 0.05.

Panel DNA sequencing was performed on a subset of patients from the discovery cohort (*n* = 156) to identify differences between TMS3 and the other subtypes. In this sub‐cohort, there were 59 TMS0 patients, 57 TMS1 patients, 28 TMS2 patients, and 12 TMS3 patients. Oncoplots show the top 10 mutated genes within each TMS classification (Figure 4A–D). When TMS0 and TMS3 were compared by Fisher's exact test, there was a significantly higher rate of *TP53* mutation in TMS3 cases (*p* = 0.0026) (Figure 4E). *ARID1A*, *RPL22*, and *MAP2K1* mutations were enriched in TMS0, but these did not reach statistical significance. When TMS1 and TMS3 cases were compared, *ARID1A* mutations were more frequently detected in TMS1 versus TMS3 cases (*p* = 0.029) (Figure 4F). *MAP2K1* and *SF3B1* were more frequently mutated in TMS3 tumours but these did not reach statistical significance (Figure 4F). Fisher's exact tests revealed no significant differences between TMS2 and TMS3, but *ARID1A* was mutated in 24% of TMS2 cases compared to 0% of TMS3 cases (Figure 4G). *MAP2K1* was not mutated in any TMS2 cases; however, mutations were detected in 7% of TMS3 tumours.

When full‐transcriptome sequencing was performed on a subset of patients (*n* = 59) from the discovery cohort using TempO‐Seq, underlying differences in gene expression were identified between TMS3 and other classifications (supplementary material, Figure S7). Interestingly, when TMS3 were compared to TMS0 patients, there were no significantly differentially expressed genes or pathways.

**Figure 5:**
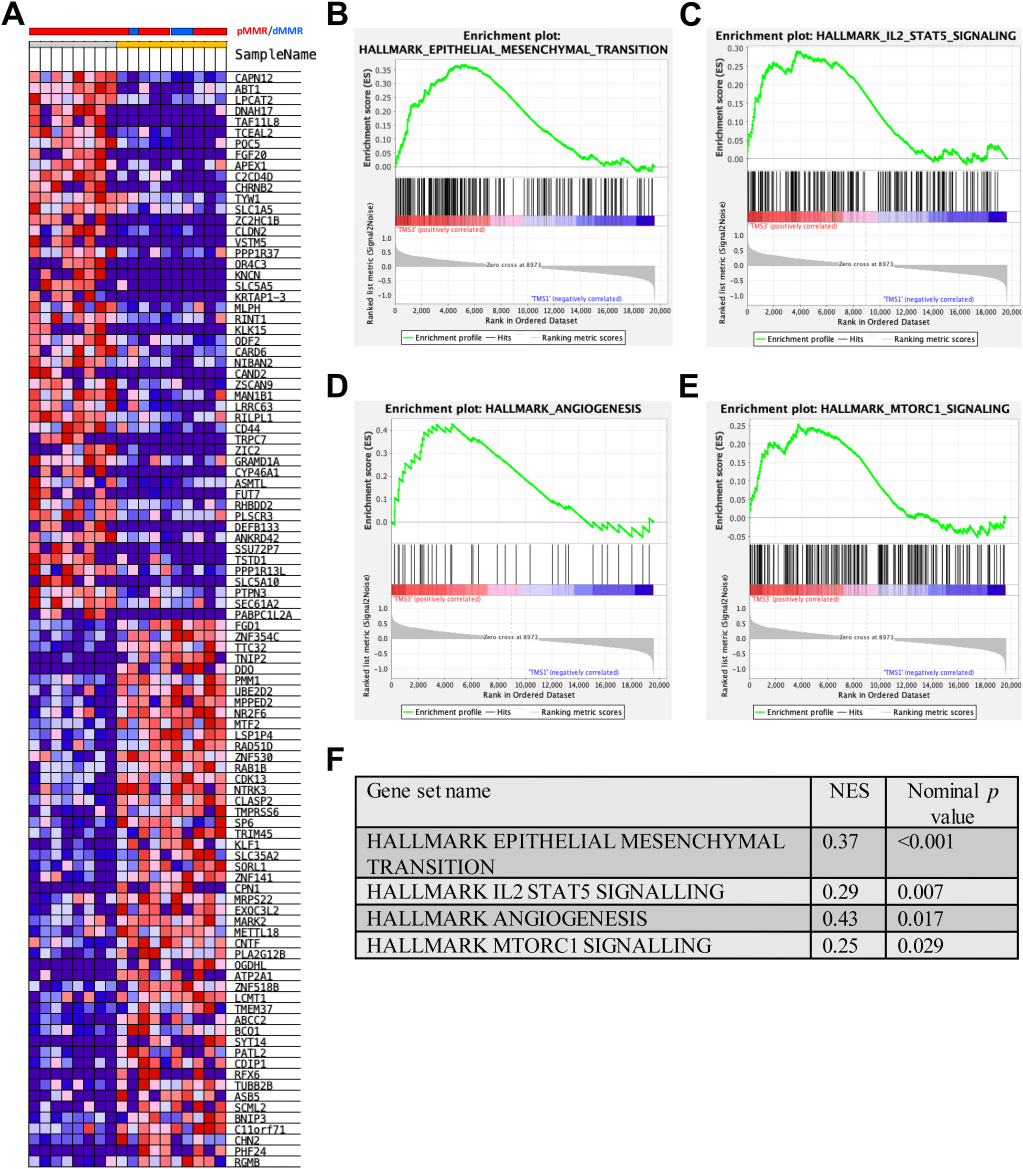
GEA of TMS3 versus TMS1. (A) Heatmap of hierarchical clustering of the top 100 most differentially expressed genes between TMS3 (grey) and TMS1 (amber) phenotypes from GSEA software (h.all.v7.5.1.symbols.gmt database). (B–F) Enrichment plots showing Hallmark signalling pathways expressed at higher levels in TMS3 versus TMS1 cases, EMT, IL2 STAT5, angiogenesis, and MTORC1.

When TMS3 cases were compared to TMS1 cases there were clear patterns of differential gene expression (Figure 5A). GSEA demonstrated enriched disease progression and metastasis‐related signalling in TMS3 compared to TMS1. The analysis showed upregulation of hallmark pathways EMT [enrichment score (ES) = 0.37, nominal *p* < 0.001], IL2 STAT5 signalling (ES = 0.29, nominal *p* = 0.007), angiogenesis (ES = 0.43, nominal *p* = 0.017), and MTORC1 signalling (ES = 0.25, nominal *p* = 0.029) (Figure 5B–F).

Subsequently, when TMS3 cases were compared to TMS2 cases there were clear patterns of differential gene expression (supplementary material, Figure S8A). GSEA showed higher expression of genes associated with hallmarks E2F pathway (ES = 0.24, nominal *p* = 0.027) and interferon gamma response (ES = 0.24, nominal *p* = 0.045) (supplementary material, Figure S8B,C). These genetic pathways are associated with driving poor outcomes in CRC which further confirms the poor prognostic nature of the TMS3 subtype.

Given the association with TMS and immune infiltrates from Figure 3, gene expression data were utilised to determine any association between TMS and immune signature. GSEA was performed to identify the significant immune‐related signalling pathways enriched within TMS3 tumours compared to the other classifications. According to the findings, patients with TMS3 were enriched for genes involved in the dysfunction of T cells (ES = 0.36, nominal *p* < 0.0001) and B‐lymphocytes (ES = 0.32 nominal *p* < 0.001). Interestingly, the upregulation of genes related to T regulatory cells (ES = 0.31 nominal *p* < 0.001) and upregulation of macrophage signatures (ES = 0.31 nominal *p* < 0.001) were observed when TMS3 was compared to other classifications (supplementary material, Figure S9A–F). These results suggest an immunosuppressive and evasive microenvironment within TMS3 which corroborate data from the protein level in Figure 3.

## Discussion

CRCs are heterogenous, which has resulted in the development of molecular classification systems to segregate patients into prognostic and predictive groups. Subtyping methods including CMS and CRIS have vastly increased the depth of our understanding of CRC disease; however, they rely on complex genomic and transcriptomic techniques to segregate disease which are not yet feasible for translation to routine diagnostics. Histopathological scoring methods such as GMS and TB are a simpler way of subtyping patients to predict prognosis, but there is limited research to date on how these could be used to determine optimal therapeutic regimes.

In this study, the combination of GMS and TB was established as a more powerful prognostic marker than the existing CRC classifications. Across two large independent retrospective patient cohorts, after patient exclusion in a discovery (*n* = 631) and a validation CRC cohort (*n* = 605), TMS was independently able to predict patient outcomes. Given the clinically translatable nature of performing TMS from a single H&E full tumour section, this represents an exciting prognostic tool which could be easily and quickly adopted into routine pathology reporting. Our findings show that patients classified as immune phenotype TMS0 had the best survival outcomes in both cohorts when compared to all other TMS classifications. This is not surprising, as many previous studies have shown the density of inflammatory infiltrate to be a major factor in disease outcomes [22]. In contrast, patients classified as invasive subtype TMS3 with an immunologically cold TME with high stromal invasion and the presence of tumour buds extravasating away from the bulk tumour were shown to have the worst outcomes.

TMS was independently prognostic from the previously validated histopathological subtyping method GMS. The percentage of TMS3 patients alive at 5‐years post‐surgery was only 37% compared to 41% of patients classified as the poorest prognostic GMS subtype GMS2 in the discovery cohort. In the validation cohort, the difference was more pronounced, with the 5‐year survival of TMS3 patients 31% versus 47% of GMS2 patients.

Similar results were demonstrated in both discovery and validation cohorts, confirming the poor prognostic value of the TMS3 subtype. Multivariate Cox regression survival analysis also showed that TMS was independently prognostic. These findings confirm the powerful value of TMS to stratify patients based on a single H&E‐stained section.

Interestingly, TMS was more prognostic in rectal than in colonic tumours. This was validated across both cohorts, driven by profoundly reduced survival times in the TMS3 populations. This could have important clinical implications due to differences in the tumour biology depending on tumour subsite and disparity in treatment options available to colon versus rectal patients. For example, rectal cancer patients are often treated with neoadjuvant radiotherapy/chemoradiotherapy, and there is emerging evidence across other tumour types that exposure to radiation can drive invasion and metastases in some cases. This highlights the importance of future studies to investigate TMS in a radiotherapy‐treated rectal cancer patient cohort. Future work could also include assessing the prognostic power of TMS within other clinically relevant subgroups such as TNM stages and relative to *KRAS*/*BRAF* mutational status.

In addition to the utility of TMS as a prognostic marker we also investigated the association with immune infiltrates to gain insight into potential mechanisms underpinning the histopathological phenotypes. There was significantly decreased influx of CD3+ cells in TMS3 in both cohorts. It is well established that T lymphocyte infiltration is associated with better prognosis in CRC, and immunologically cold tumours are more aggressive [23, 24]. Immunoscore® uses the ratio of CD3+ and CD8+ T lymphocytes at the tumour invasive edge to determine patient prognosis, with high CD8+ (cytotoxic) cells indicative of good outcome [25]. Future research could include investigating any association between Immunoscore® and TMS to explore the reduced CD3+ counts in TMS3 further. These data could lead us to hypothesise that TMS3 tumours may be unresponsive to checkpoint inhibitors and further work could include investigating checkpoint protein expression relative to TMS. Interestingly, there were no differences in gene expression identified between immune‐rich TMS0 and invasive TMS3 subgroups. This may be due to using bulk RNA sequencing which would not enable interrogation of gene expression in different compartments of the TME. Therefore, future work could include utilising spatial transcriptomic technology to identify changes in the stromal, tumour, and immune regions of interest.

To identify optimal therapeutic approaches and understand the biological characteristics underpinning each TMS classification, genomic and transcriptomic analyses were performed to establish the mutational landscape and gene expression profiles of each TMS. In the discovery cohort (*n* = 59), bulk transcriptomic data revealed TMS3 was enriched for hallmark signalling pathways such as EMT, IL2/STAT5, angiogenesis, and MTORC1. These are well established as being related to disease progression and metastasis in the literature and highlight potential mechanisms underlying the poor prognostic nature of TMS3 [26].

There are a number of therapeutic approaches which could be utilised to inhibit these signalling proteins and potentially reverse the TMS3 phenotype. For example, inhibitors of EMT‐related protein ZEB1 have shown therapeutic potential in ovarian carcinoma *in vitro* [27]. Similarly, an inhibitor of TWIST1 signalling had anti‐tumour activity in patient‐derived xenograft models of non‐small lung cancer [28]. In terms of clinically approved drugs which target EMT‐related proteins, anti‐EGFR therapeutic cetuximab is currently utilised for metastatic CRC [29]. Future work should include establishing the potential for cetuximab in TMS3 tumours specifically across stage I–III disease. Similarly, anti‐angiogenic drug bevacizumab, which targets VEGF, is also approved for clinical use in advanced stage CRC and could be investigated for TMS3 patients due to the enrichment of the hallmark angiogenesis pathway [30].

Prior to identification of optimal therapeutic regimens for each subgroup, the TMS already provides clinical value. TMS is independently prognostic from GMS and identified a group of patients within GMS2 with even worse clinical outcomes. Although CMS subtypes classify CRC patients into biologically distinct subgroups, CMS requires complex and costly RNA sequencing and bioinformatics approaches to segregate patient disease. These, therefore, cannot be utilised clinically within an appropriate timeframe. TMS, however, can be performed using a single H&E‐stained tumour section which is already being done in the clinical setting. Initially, this could be performed manually by a pathologist; however, work is in progress to digitise each of the TMS components using computational pathology, which would improve the speed and reproducibility of scoring.

A potential limitation of the TMS is the presence of pseudo‐buds within tumours scored as high for TB. Pseudo‐buds have a similar morphology to true tumour buds but can be distinguished based on the proliferation status of budding cells (by Ki67 immunohistochemical staining) [31]. Pseudo‐budding may result in patients being assigned to TMS2 or TMS3 instead of TMS1 or TMS2, respectively, and therefore incorporation of a Ki67‐stained resection to score TB for TMS should be considered.

In conclusion, TMS is a novel histopathological method of segregating CRC patients to independently predict clinical outcome. The phenotypic differences observed in poor‐prognosis group TMS3 are clearly underpinned by distinct targetable biology. Further research is required to validate factors driving the observed phenotypes and determine optimal therapeutic approaches for patients classified as each TMS.

## Author contributions statement

PH, KP contributed to conceptualisation, data curation, formal analysis, investigation, methodology and project administration. PA and HvW were involved in data curation and methodology. AR performed data curation, supervision and methodology. JI contributed to data curation and investigation. JH contributed to conceptualisation, supervision, resources and funding acquisition. DA contributed to data curation, supervision and resources. NM contributed to supervision, resources and validation. JP contributed to conceptualisation, supervision, methodology, data curation and resources. CR contributed to supervision and resources. CT contributed to supervision and funding acquisition. DM was involved in conceptualisation, resources and supervision. JE contributed to conceptualisation, supervision, funding acquisition, validation, methodology, investigation and project administration. All authors were involved in writing the original draft, review and editing of the paper.

## Supporting information


**Figure S1.** Molecular and phenotypic classifications of CRC
**Figure S2.** The relationship between CMS, GMS, and TMS
**Figure S3.** Patient inclusion from the discovery cohort
**Figure S4.** Patient inclusion from the validation cohort
**Figure S5.** Association between TMS and clinicopathological characteristics
**Figure S6.** TMS in relation to MMR status
**Figure S7.** TMS3 is underlined by a different gene expression profile
**Figure S8.** GSEA of TMS3 versus TMS2
**Figure S9.** GSEA of TMS3 versus other TMS classifications based on immunogenic gene signature sets
**Table S1.** List of genes included in mutational profiling
**Table S2.** Univariate and multivariate Cox regression analyses for TMS and immune infiltrates in cohort 1
**Table S3.** Univariate and multivariate Cox regression analyses for TMS and immune infiltrates in cohort 2

## Data Availability

Data for cohort 1 are held within the Glasgow and Clyde Safe Haven (GSH/18/ON007). Data from cohort 2 are held within the Glasgow and Clyde Safe Haven (GSH21ON009). TempO‐Seq data can be accessed at ArrayExpress at E‐MTAB‐13077.
