# Supplementary material for: Histopathological tumour microenvironment score independently predicts outcome in primary operable colorectal cancer
Source: J Pathol Clin Res. 2024 Apr 22;10(3):e12374. doi: 10.1002/2056-4538.12374 (PMC11035902; doi:10.1002/2056-4538.12374)
Supplement: Supplementary file 1 — Figure S1. Molecular and phenotypic classifications of CRC Figure S2. The relationship between CMS, GMS, and TMS Figure S3. Patient inclusion from the discovery cohort Figure S4. Patient inclusion from the validation cohort Figure S5. Association between TMS and clinicopathological characteristics Figure S6. TMS in relation to MMR status Figure S7. TMS3 is underlined by a different gene expression profile Figure S8. GSEA of TMS3 versus TMS2 Figure S9. GSEA of TMS3 versus other TMS classifications based on immunogenic gene signature sets Table S1. List of genes included in mutational profiling Table S2. Univariate and multivariate Cox regression analyses for TMS and immune infiltrates in cohort 1 Table S3. Univariate and multivariate Cox regression analyses for TMS and immune infiltrates in cohort 2 [file CJP2-10-e12374-s001.pdf]

Histopathological tumour microenvironment score independently predicts outcome in primary operable colorectal cancer

P Hatthakarnkul, K Pennel *et al,*. *J Pathol Clin Res,* https://doi.org/10.1002/2056-4538.12374

Supplementary Figures S1–S9
Supplementary Tables S1–S3

A

CMS	Immune Infiltrate	MSI status	Mutations	Signatures
1-Immune	High	High	BRAF	Immunogenic
2- Canonical	Any	Any	TP53	WNT and MYC activation
3- Metabolic	Any	Mix	KRAS	Metabolic deregulation
4- Mesenchymal	Low	Any	NA	TGF-β activation, angiogenesis

B

GMS	KM Grade	TSP
0	High (2/3)	Any
1	Low (0/1)	Low (<50%)
2	Low (0/1)	High (>50%)

C

TMS	KM Grade	TSP	Tumour budding
0	High (2/3)	Any	Any
1	Low (0/1)	Low (<50%)	Low
2	Low (0/1)	One High	
3	Low (0/1)	High (>50%)	High

Figure S1. Molecular and phenotypic classifications of colorectal cancer. The classifications used to determine (A) Consensus Molecular Subtype (CMS), (B) Glasgow Microenvironment Score (GMS) and (C) Tumour Microenvironment Score (TMS).

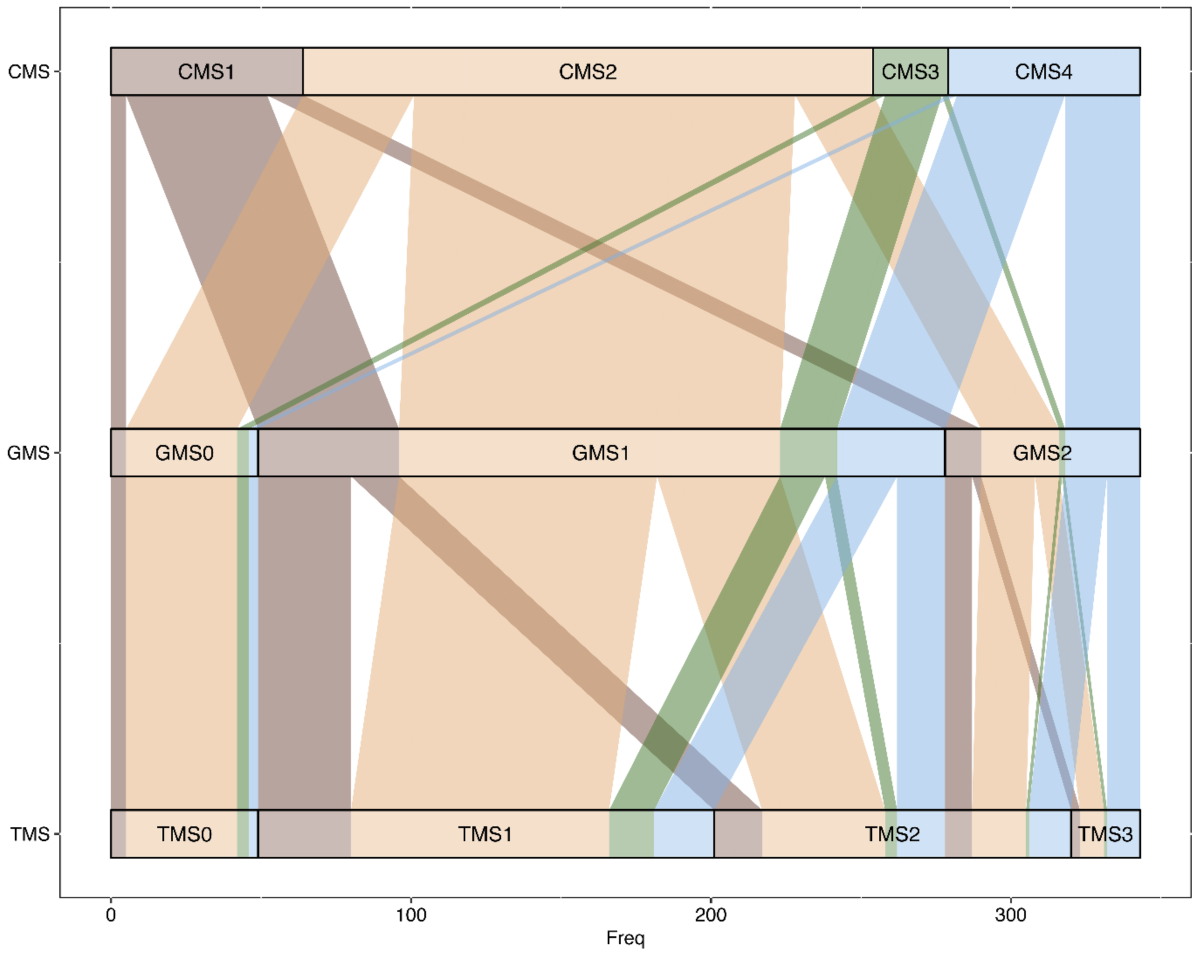

Figure S2. The relationship between CMS, GMS and TMS. Alluvial plot showing the overlap between patients categorised as CMS, GMS and TMS.

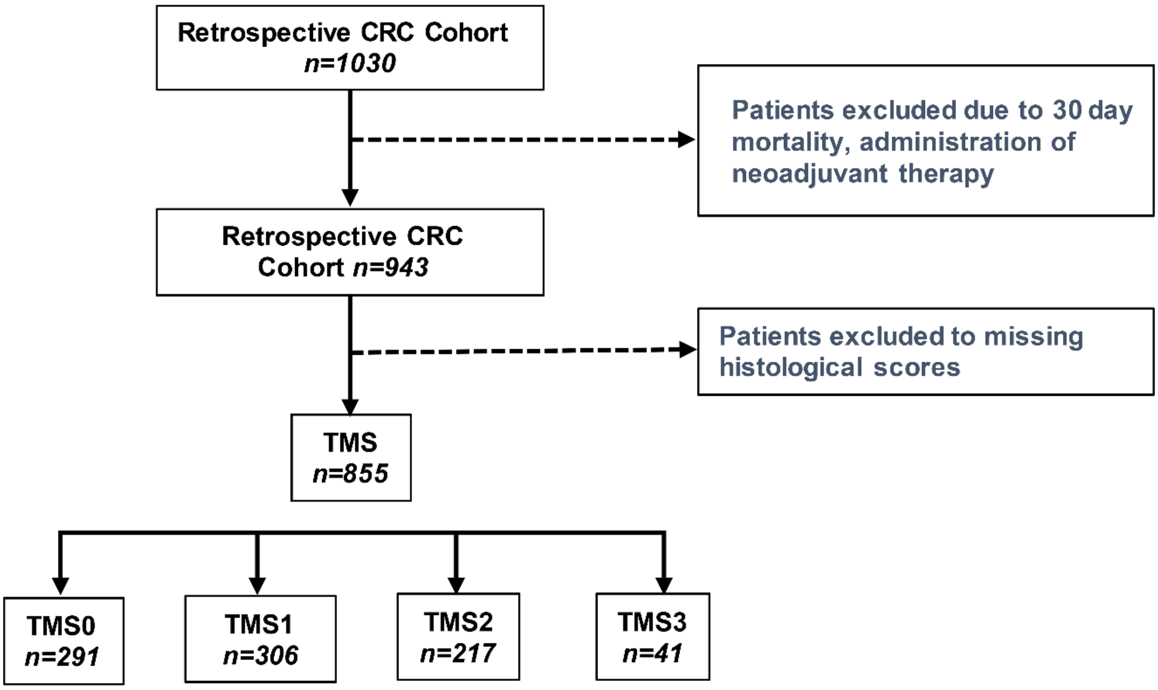

Figure S3. Patient inclusion from the discovery cohort. Consort diagram highlighting the number of patients included in downstream analysis following application of exclusion criteria.

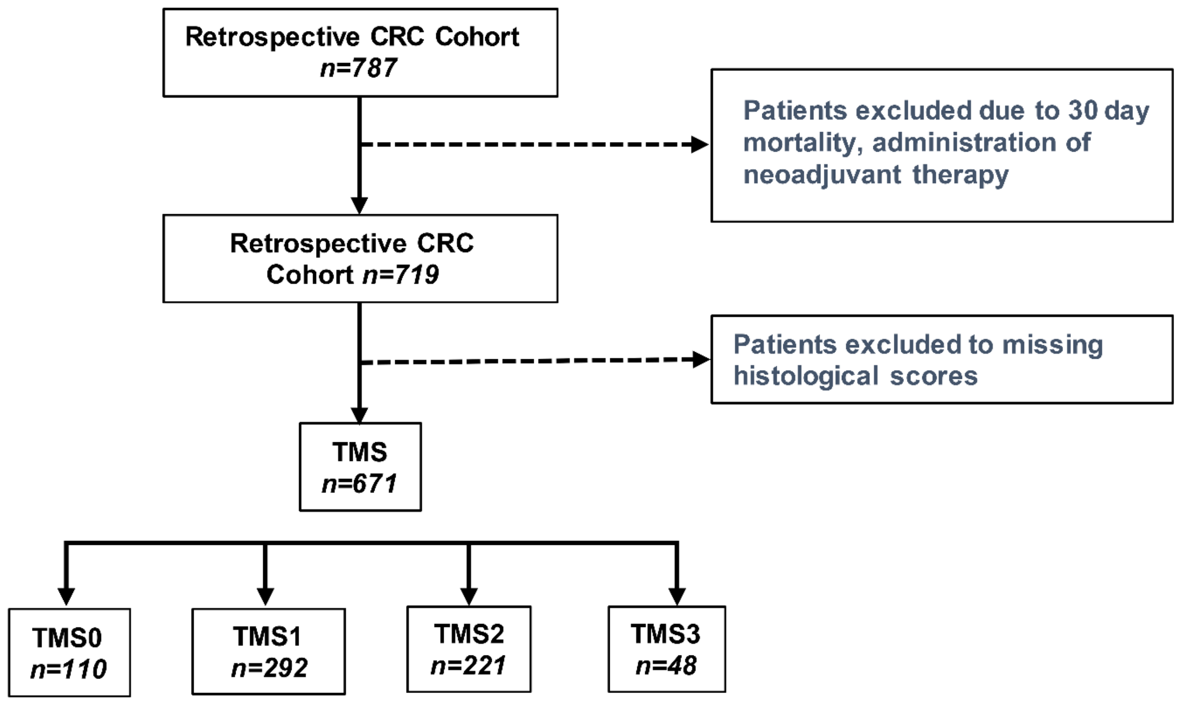

Figure S4. Patient inclusion from the validation cohort. Consort diagram highlighting the number of patients included in downstream analysis following application of exclusion criteria.

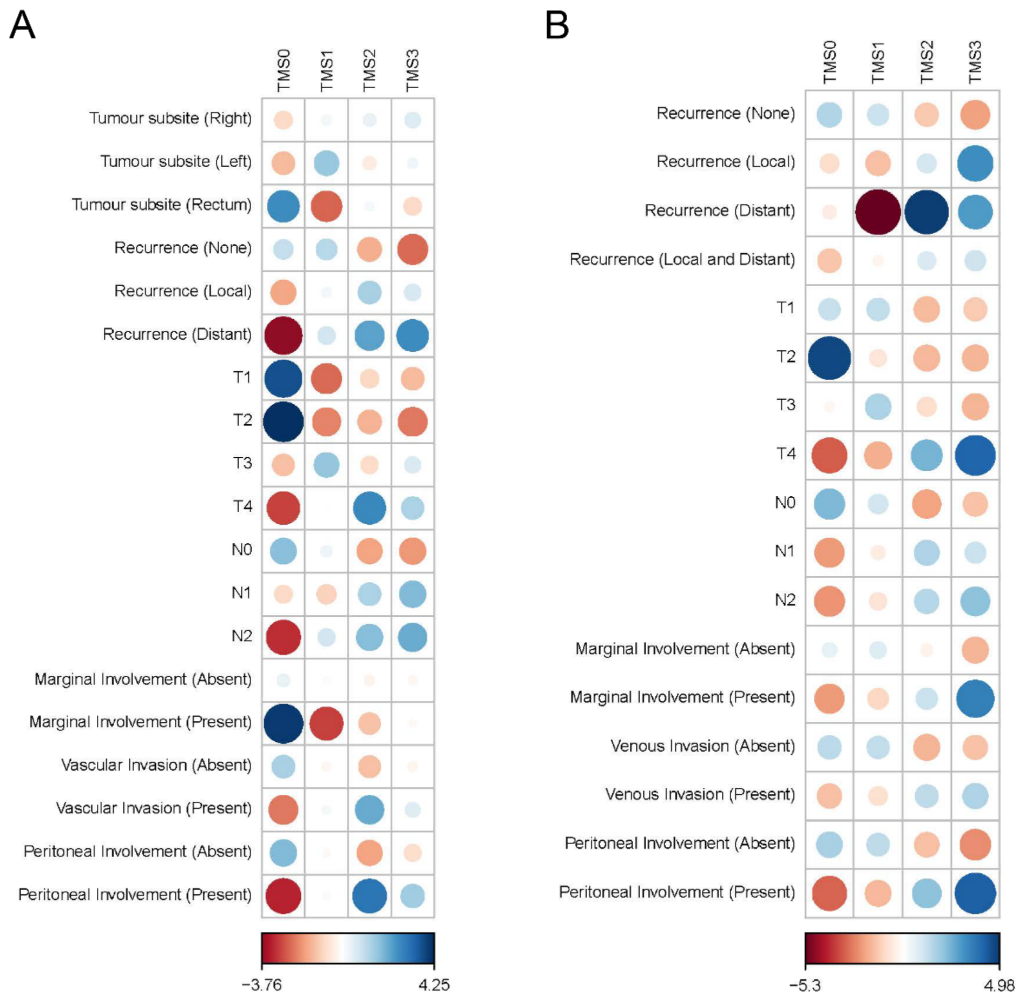

Figure S5. Association between TMS and clinicopathological characteristics. Correlation plots showing the association between TMS and factors significant upon Chi-squared analysis in (A) the discovery and (B) the validation cohorts. Dot size and colours reflect Z score, with red indicative of positive enrichment and blue negative enrichment.

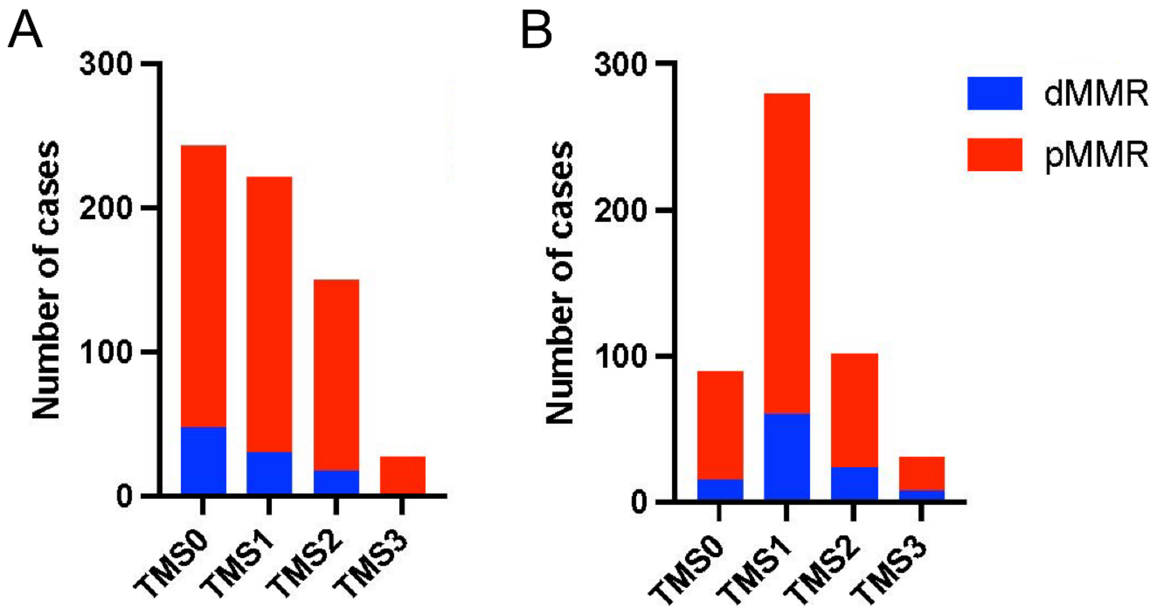

Figure S6. TMS in relation to MMR status. Bar charts showing the distribution of dMMR and pMMR cases across each TMS subgroup.

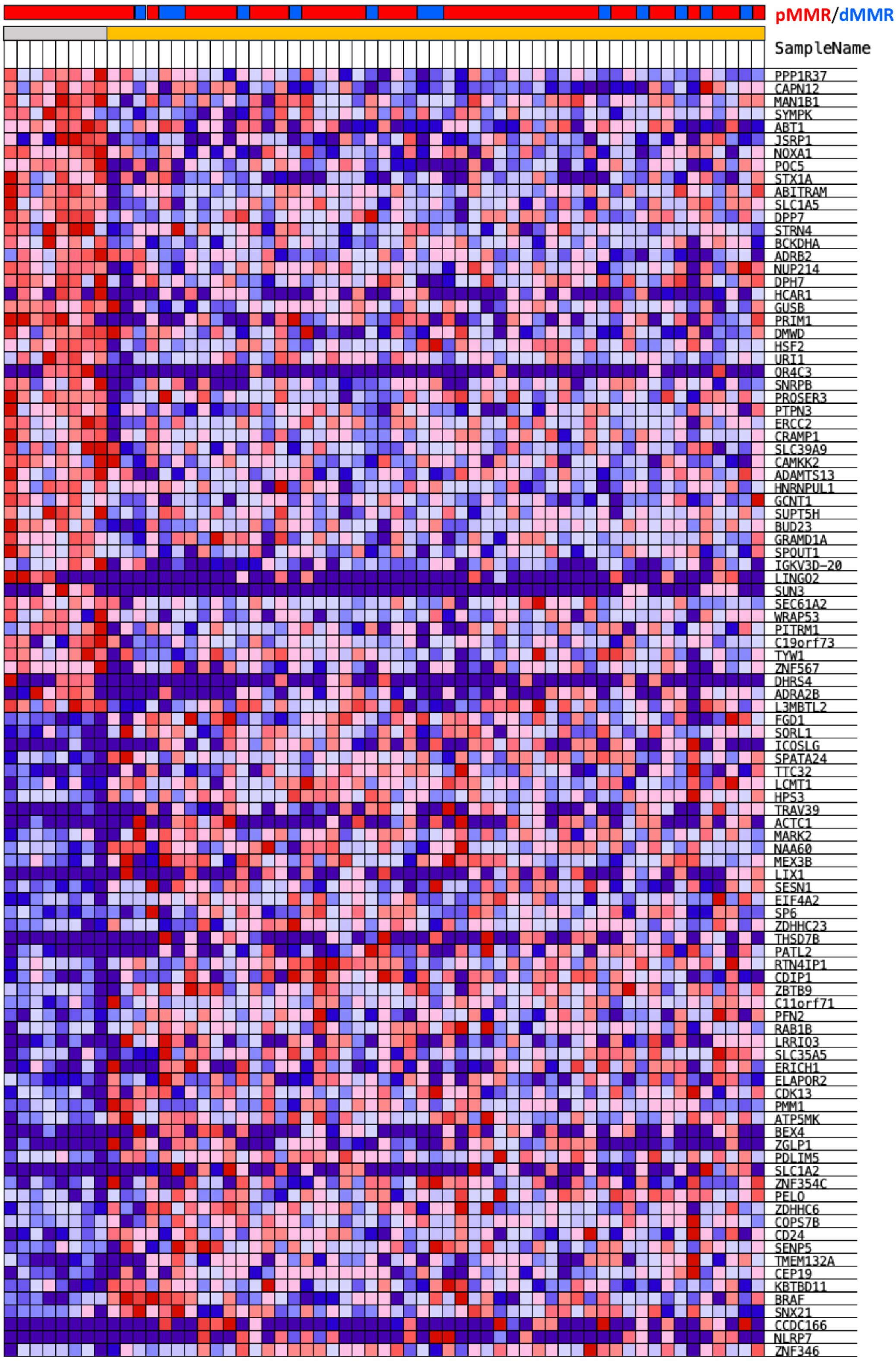

Figure S7. TMS3 is underlined by a different gene expression profile. Heatmap showing the top differentially expressed genes between TMS3 and other TMS classifications in a subset of the discovery cohort. Grey represents TMS3 and yellow represents other TMS groups.

A B C

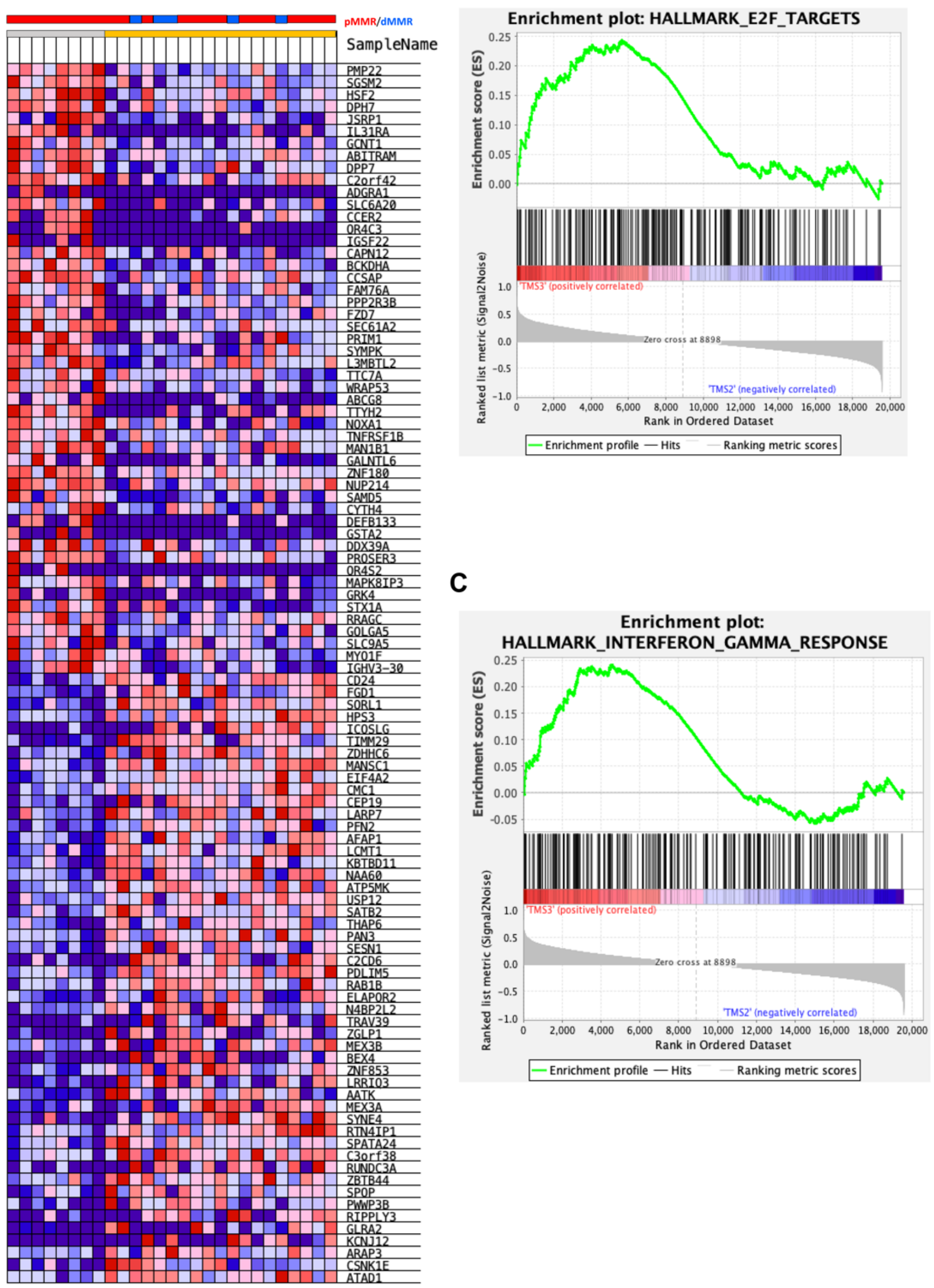

D

Gene set name	NES	Nominal P value
E2F TARGETS	0.24	0.027
INTERFERON GAMMA RESPONSE	0.24	0.045

Figure S8. Gene set enrichment analysis of TMS3 versus TMS2. (A) Heatmap showing the top differentially expressed genes between TMS3 and TMS2 classifications. Grey represents TMS3 and yellow represents TMS2. (B, C) Enrichment plots showing Hallmark signalling pathways expressed at higher levels in TMS3 versus TMS2 cases, E2F and Interferon gamma response.

A

Gene set number	NES	Nominal P value
GSE22886	0.36	<0.001
GSE10325	0.33	<0.001
GSE21360	0.31	<0.001
GSE18893	0.31	<0.001
GSE22935	0.35	<0.001

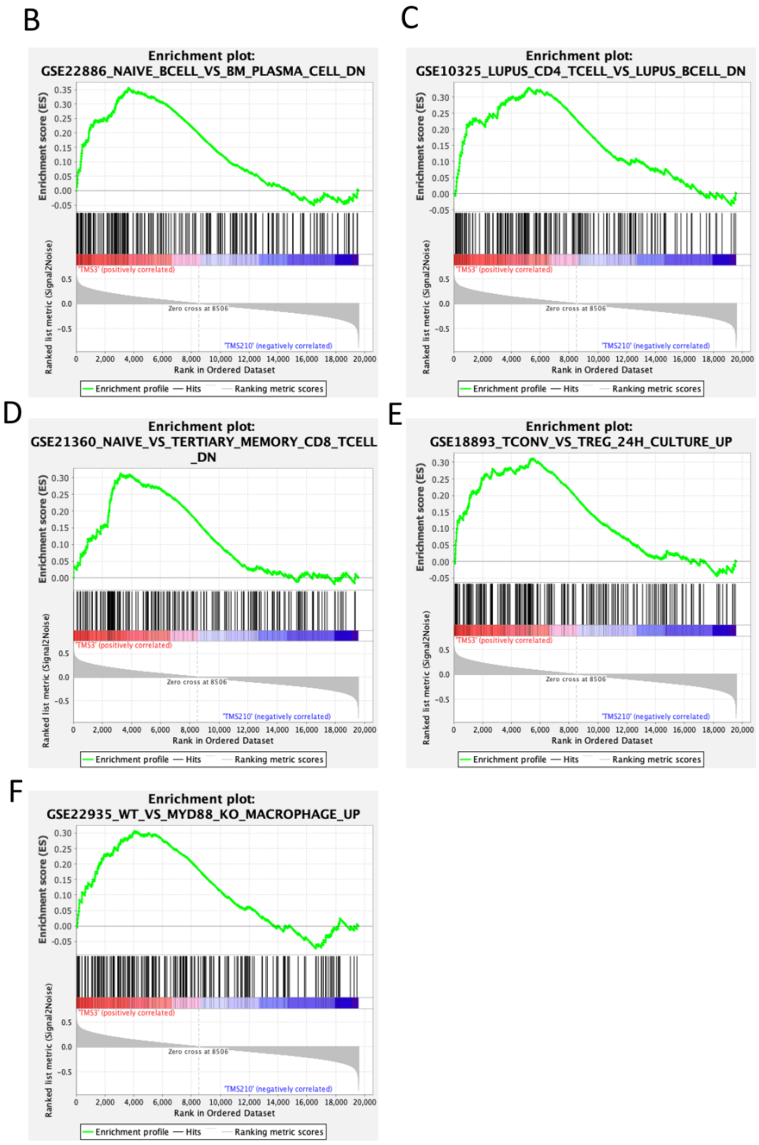

Figure S9. Gene set enrichment analysis of TMS3 versus other TMS classifications based on immunogenic gene signature sets. (A) Enrichment results for TMS3 versus other classifications using immune related gene sets. (B-F) Enrichment plots showing immunogenic gene signature sets expressed at higher levels in TMS3 compared to other phenotypes including (B) naïve B cells versus plasma cells, (C) CD4+ T cells versus B cells, (D) naïve versus tertiary memory cytotoxic T cells, (E) conventional versus regulatory T cells and (F) conventional versus MYD88 knock out macrophages.

Table S1. Panel of genes included in mutational profiling.

PIK3CA	*EGFR*	*SF3B1*	*JAK2*	*BAP1*	*AKT2*	*FLT4*	*ABL2*
FBXW7	*SMO*	*NOTCH3*	*CDKN2A*	*JAK1*	*NF2*	*CYP2D6*	*INPP4B*
APC	*WT1*	*CHEK2*	*RET*	*RB1*	*IDH1*	*LAMA2*	*PDGFRB*
BRAF	*ATM*	*AMER1*	*FGFR2*	*RUNX1*	*ZNF395*	*TRRAP*	*DDR1*
TP53	*KMT2A*	*MTOR*	*CASP5*	*NTRK1*	*MUTYH*	*KDM6A*	*TAS2R38*
SMARCA4	*ERBB3*	*NOTCH4*	*MDM2*	*CACNA1E*	*JUN*	*ABCC2*	*ABL1*
PPP2R1A	*TMBIM4*	*NOTCH1*	*MAX*	*DAXX*	*NFE2L2*	*CHD7*	*RARB*
AR	*POLE*	*FGFR3*	*DICER1*	*CDKN2B*	*VHL*	*SRC*	*MYD88*
KRAS	*BRCA2*	*EP300*	*BRCA1*	*CCND1*	*HGF*	*NELL2*	*RXRB*
MSH6	*B2M*	*PTCH1*	*PTEN*	*STK11*	*GNAQ*	*UGT1A6*	*ABCB1*
ARID2	*IDH2*	*CTCF*	*FGFR1*	*EZH2*	*MAP2K1*	*ESR1*	*RARA*
ERBB2	*BLM*	*PALB2*	*GATA3*	*CDKN1B*	*MAPK1*	*DDX3X*	*DDR2*
RAF1	*TSC2*	*NOTCH2*	*DNMT3A*	*KIT*	*RHOA*	*SHOC2*	*LTK*
RPL22	*CREBBP*	*ATRX*	*ALK*	*MET*	*PHF6*	*ERG*	*NPM1*
TAF1B	*CIITA*	*IPP*	*NF1*	*CDK4*	*HRAS*	*CEBPA*	*CYP19A1*
MSH2	*RNF43*	*ASXL1*	*STAG2*	*PTPN11*	*RPL5*	*CYP2A6*	*SLC31A1*
COBLL1	*MAP2K2*	*GNAS*	*SMAD4*	*MAP2K4*	*RAD21*	*AKT3*	*FSTL5*
TGFBR2	*JAK3*	*AIM2*	*RAD50*	*CDK12*	*H3F3A*	*MED13*	*CYP2C19*
CTNNB1	*CCNE1*	*CCDC150*	*TSC1*	*CDKN1A*	*RAC1*	*MST1R*	*CBL*
POLQ	*TEAD2*	*ERBB4*	*KDR*	*NBN*	*CD274*	*SLC22A1*	*RXRA*
ASTE1	*SLC23A2*	*MLH1*	*FGFR4*	*PIK3CB*	*TET2*	*MPL*	
ATR	*ARAF*	*SETD2*	*QKI*	*GNA11*	*SOS1*	*HNF1A*	
PIK3R1	*ARID1A*	*PDGFRA*	*MEN1*	*AURKA*	*CSF1R*	*YES1*	
TTK	*NRAS*	*MAP3K1*	*SPOP*	*HIST1H3B*	*SLCO1B1*	*IL2RA*	
ARID1B	*PBRM1*	*ROS1*	*AKT1*	*MYC*	*FLT1*	*TYK2*	

Table S2. Univariate and multivariate cox regression analyses for TMS and immune infiltrates in cohort 1.

Discovery cohort

**Clinicopathological characteristics**	**Univariable analysis**		**Multivariate analysis**	
	**Hazard ratio (95% CI)**	***p***	**Hazard ratio (95% CI)**	***p***
**TMS**	1.615 (1.415-1.843)	<0.001	1.667 (1.387-2.003)	<0.001
**CD3**	0.573 (0.446-0.737)	<0.001	0.789 (0.550-1.132)	0.198
**CD8**	0.598 (0.455-0.787)	<0.001	0.824 (0.548-1.241)	0.354
**FOXP3**	0.515 (0.378-0.703)	<0.001	0.699 (0.470-1.040)	0.077
**CD68**	1.465 (1.091-1.967)	0.011	1.383 (0.972-1.968)	0.072

Table S3. Univariate and multivariate cox regression analyses for TMS and immune infiltrates in cohort 2.

Validation cohort

**Clinicopathological characteristics**	**Univariable analysis**		**Multivariate analysis**	
	**Hazard ratio (95% CI)**	***p***	**Hazard ratio (95% CI)**	***p***
**TMS**	1.514 (1.351-1.697)	<0.001	1.413 (1.235-1.635)	<0.001
**CD3**	0.670 (0.492-0.913)	0.011	0.746 (0.524-1.060)	0.102
**CD3CD8**	0.533 (0.355-0.801)	0.002	0.581 (0.360-0.936)	0.026
**CD3FOXP3**	1.482 (1.068-2.056)	0.010	1.249 (0.863-1.809)	0.239
**CD68**	2.585 (1.606-4.159)	<0.001	2.106 (1.186-3.739)	0.011
